# Improvement in XIa Selectivity of Snake Venom Peptide Analogue BF9-N17K Using P2′ Amino Acid Replacements

**DOI:** 10.3390/toxins17010023

**Published:** 2025-01-05

**Authors:** Li Ding, Zhiping Zhai, Tianxiang Qin, Yuexi Lin, Zhicheng Shuang, Fang Sun, Chenhu Qin, Hongyi Luo, Wen Zhu, Xiangdong Ye, Zongyun Chen, Xudong Luo

**Affiliations:** 1 Institute of Biomedicine, Hubei Key Laboratory of Embryonic Stem Cell Research, College of Basic Medicine, Hubei University of Medicine, Shiyan 442000, China; dl2168@163.com (L.D.); zzp13081653526@163.com (Z.Z.); lyx1346211@163.com (Y.L.); shuangzc1999@126.com (Z.S.); 2016202040055@whu.edu.cn (F.S.); 2016202040025@whu.edu.cn (C.Q.); 2016202040022@whu.edu.cn (H.L.); zhuwen0712@126.com (W.Z.); yexiangdong@hbmu.edu.cn (X.Y.); 2 Department of Clinical Laboratory, Dongfeng Hospital, Hubei University of Medicine, Shiyan 442000, China; 3 Hubei Key Laboratory of Wudang Local Chinese Medicine Research, Hubei University of Medicine, Shiyan 442000, China

**Keywords:** XIa inhibitor, plasmin inhibitor, snake venom peptide, Kunitz-type, P2′ site

## Abstract

Coagulation factor XIa is a new serine-protease family drug target for next-generation anticoagulants. With the snake venom Kunitz-type peptide BF9 as the scaffold, we obtained a highly active XIa inhibitor BF9-N17K in our previous work, but it also inhibited the hemostatic target plasmin. Here, in order to enhance the selectivity of BF9-N17K toward XIa, four mutants, BF9-N17K-L19A, BF9-N17K-L19S, BF9-N17K-L19D, and BF9-N17K-L19K, were further designed using the P2′ amino acid classification scanning strategy. The anticoagulation assay showed that the four P2′ single-point mutants still had apparent inhibitory anticoagulation activity that selectively inhibited the human intrinsic coagulation pathway and had no influence on the extrinsic coagulation pathway or common coagulation pathway, which indicated that the single-point mutants had minimal effects on the anticoagulation activity of BF9-N17K. Interestingly, the enzyme inhibitor assay experiments showed that the XIa and plasmin inhibitory activities were significantly changed by the P2′ amino acid replacements. The XIa inhibitory activity of BF9-N17K-L19D was apparently enhanced, with an IC_50_ of 19.28 ± 2.53 nM, and its plasmin inhibitory was significantly weakened, with an IC_50_ of 459.33 ± 337.40 nM. BF9-N17K-L19K was the opposite to BF9-N17K-L19D, which had enhanced plasmin inhibitory activity and reduced XIa inhibitory activity. For BF9-N17K-L19A and BF9-N17K-L19S, no apparent changes were found in the serine protease inhibitory activity, and they had similar XIa and plasmin inhibitory activities to the template peptide BF9-N17K. These results suggested that the characteristics of the charge of the P2′ site might be associated with the drug selectivity between the anticoagulant target XIa and hemostatic target plasmin. In addition, according to the molecular diversity and sequence conservation, a common motif GR/PCR/KA/SXIP-XYGGC is proposed in the XIa-inhibitory Kunitz-type peptides, which might provide a new clue for further peptide engineering. In conclusion, through P2′ amino acid classification scanning with the snake venom Kunitz-type peptide scaffold, a new potent and selective XIa inhibitor, BF9-N17K-L19D, was discovered, which provides a new XIa-targeting lead drug template for the treatment of thrombotic-related diseases.

## 1. Introduction

Blood coagulation has physiological significance in preventing bleeding in the body, which is mainly mediated by the tissue factor (TF) and the activation of the extrinsic coagulation pathway [1,2]. Additionally, blood coagulation also can be initiated by the contract activation pathway, which is mainly mediated by exposure to negatively charged surfaces, such as heparin, collagen, polyphosphate, and nucleic acid; over-sulfated chondroitin sulfate; amyloid β peptides 1–42; and exogenous negatively charged surfaces [3,4,5]. Because the intrinsic coagulation pathway can be activated by many immune- and inflammatory-related molecules and because the intrinsic coagulation-associated coagulation factors XIa, XIIa, and PK do not cause apparent bleeding due to their deficiencies [3,6,7], the intrinsic coagulation pathway is not thought to play the main role in preventing bleeding in the physiological state. So, the intrinsic coagulation pathway, which is associated coagulation factors and especially the coagulation factor XIa, might be inhibited to modulate the uncontrolled coagulation response and thrombosis-associated diseases [8]. In fact, Phase 2 and Phase 3 trials have shown that XIa might be used as a target in anticoagulation therapy [9,10]. The inhibition of XIa is associated with minimally increased rates of bleeding and the prevention of thromboembolic events, indicating the potential role of XIa as a therapeutic modifier as an anticoagulant of small molecules such as BAY-2433334 and BMS-986177, monoclonal antibodies MAA868 and AB023, and two antisense oligonucleotides, ISIS-416858 and ISIS-957943 [11,12]. So, coagulation factor XIa is thought to be a new serine-protease family drug target for next-generation anticoagulants, which strongly interferes with the contact activation stage of the intrinsic coagulation pathway and might have minimal effects on the physiological coagulation pathway [10,12].

Kunitz-type peptides from venomous and blood-sucking animals are natural sources of XIa peptide inhibitors [13,14,15]. Improving the specificity and exploring the common structural characteristics of these inhibitors remain challenging scientific problems, but molecular design combined with natural Kunitz-type XIa inhibitor discovery might be two effective methods to overcome these problems, using BF9 and Fasxiator from the snake *Bungarus fasciatus* [16,17,18], PN2KPI from the human protease nexin 2 [19,20], WPK5 from the Leech *Whitmania pigra* [21], DAKS1 from the snake *Deinagkistrodon acutus* [22], Ir-CPI from the tick *Ixodes Ricinus* [13], desmolaris from the vampire bat *Desmodus rotundus* [14], and some artificially designed Kunitz-type peptides Fasxiator-N17R-L19E, DX-88mut, BF9-N17K, BF9-N17R, PN2KPI-M17D, and WPK5-mut [17,21,23,24,25]. BF9 is a Kunitz-type anticoagulation peptide from snake venom with weak XIa inhibitory activity [16]. In our previous work, through P1 site amino acid classification scanning with different kinds of amino acids, such as polar amnio acids, nonpolar amino acids, acidic amino acids, and basic amino acids, a new XIa inhibitor, BF9-N17K, was discovered, which has significantly enhanced inhibitory activity toward XIa when compared to that of the wild-type template peptide BF9 [24]. However, the inhibitory activity of BF9-N17K toward plasmin, an opposite hemostatic target, was also increased, which might cause significant hemostatic side effects in real application scenarios [17,26].

Here, in order to enhance the selectivity of Kunitz-type scaffold peptide BF9-N17K from snake venom toward XIa, four BF9-N17K analogues were further designed using the P2′ site amino acid classification scanning strategy, and a new potent and selective XIa inhibitor, BF9-N17K-L19D, was found. Our work provides a new XIa-inhibitory lead drug with a snake venom Kunitz-type peptide scaffold and highlights the potential application of the P2′ site scanning strategy to enhance the selectivity of Kunitz-type peptides toward specific serine proteases.

## 2. Results

### 2.1. Molecular Design of Four Single-Point Mutants with Snake Venom Kunitz-Type Peptide Scaffold BF9-N17K

**Figure 1:**
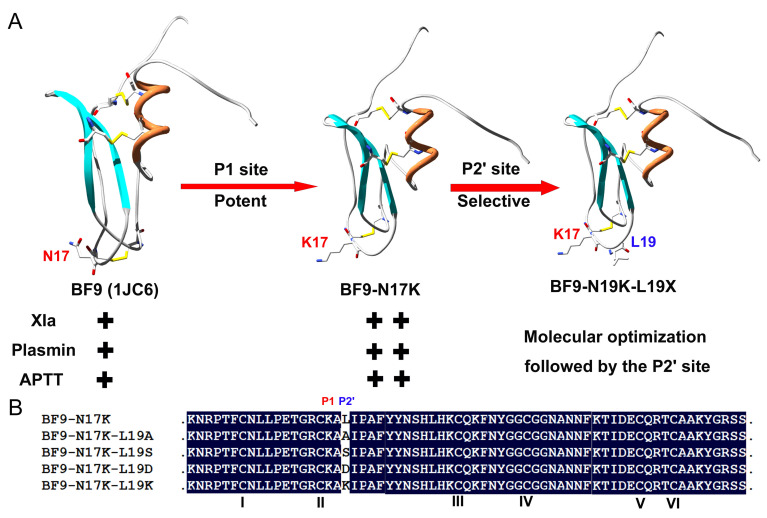
Molecular design of four BF9-N17K mutants via P2′ site scanning with different kinds of amino acids. (**A**) Snake venom Kunitz-type peptide BF9 was the template for designing new potent and selective anticoagulants targeting XIa with low plasmin inhibitory activity. + means weak activity, and ++ means strong activity. (**B**) Amino acid sequence alignments of four BF9-N17K mutants produced via P2′ site scanning with different kinds of amino acids, A, S, D, and K. I–VI showed that there are six classical cysteines in the Kunitz-type peptide BF9 and its analogues.

Kunitz-type peptides adopt a conserved pear-shaped structural scaffold that is stabilized by three conserved disulfide bridges [15]. The first functional loop of Kunitz-type peptides that corresponds to the P4-P4′ site plays a vital role in its serine protease inhibitory activity [27]. In our previous work, via P1 site scanning, we engineered a highly activity XIa inhibitor, BF9-N17K, with a weak-activity snake venom peptide BF9 as the scaffold [16,24]. However, although the natural snake venom peptide BF9 had weak activity toward plasmin, the designed BF9-N17K had enhanced plasmin inhibitory activity, which limited its application as a new anticoagulant. Here, on the basis of BF9-N17K, the P2′ site amino acid classification scanning strategy was further used to optimize its selectivity toward the coagulation factor XIa [28,29,30]. According to the basic classification of 20 amino acids, 4 representative amino acids were selected to replace the P2′ site of the template peptide BF9-N17K, which were nonpolar amino acid A, polar amino acid S, acidic amino acid D, and basic amino acid K (Figure 1). Using a similar protein expression and purification system to those of the template peptide BF9-N17K, as we have described before [16,18,24,31,32], the four newly designed peptides, BF9-N17K-L19A, BF9-N17K-L19S, BF9-N17K-L19D, and BF9-N17K-L19K, were expressed and purified successfully. High-performance liquid chromatography showed that all four engineered peptides had a single peak at about 14–16 min (Figure S1), which were collected manually and were preserved by freeze-drying after BCA quantification.

### 2.2. Anticoagulation Functional Characterization of Four BF9-N17K Mutants

**Figure 2:**
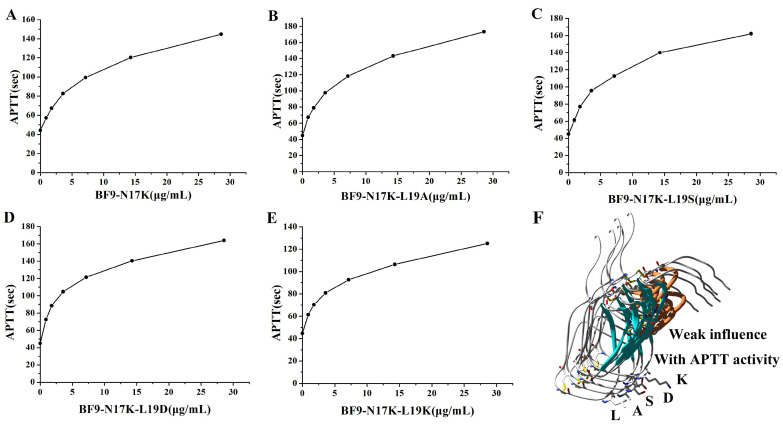
Intrinsic coagulation pathway inhibitory activity of BF9-N17K and four designed analogues tested through APTT experiments. (**A**) Intrinsic coagulation pathway inhibitory activity of BF9-N17K; (**B**) intrinsic coagulation pathway inhibitory activity of BF9-N17K-L19A; (**C**) intrinsic coagulation pathway inhibitory activity of BF9-N17K-L19S; (**D**) intrinsic coagulation pathway inhibitory activity of BF9-N17K-L19D; (**E**) intrinsic coagulation pathway inhibitory activity of BF9-N17K-L19K; (**F**) structural and intrinsic coagulation pathway inhibitory function comparison of BF9-N17K and four designed analogues.

The anticoagulation function of the four designed peptides was evaluated firstly through APTT, PT, and TT experiments. The APTT experiments showed that all four designed peptides still had apparent anticoagulation activity toward the intrinsic coagulation pathway that was similar to that of the template peptide BF9-N17K (Figure 2). This suggested that the replacement of the P2′ site with different kinds of amino acids, including the nonpolar amino acid A, polar amino acid S, acidic amino acid D, and basic amino acid K, had no apparent influence on anticoagulation through the intrinsic coagulation pathway of the Kunitz-type peptide BF9-N17K. Additionally, the PT and TT experiments showed the all four peptides showed no apparently inhibition activity toward the extrinsic coagulation pathway or the common coagulation pathway (Figures S2 and S3), which is also similar to that of the template peptide BF9-N17K. In summary, the single-point mutation of the P2′ site of BF9-N17K had minimal effects on its anticoagulation activity or anticoagulation profile.

### 2.3. Evaluation of XIa, Plasmin, and Other Serine Protease Inhibitory Activity of Four BF9-N17K Mutants

**Figure 3:**
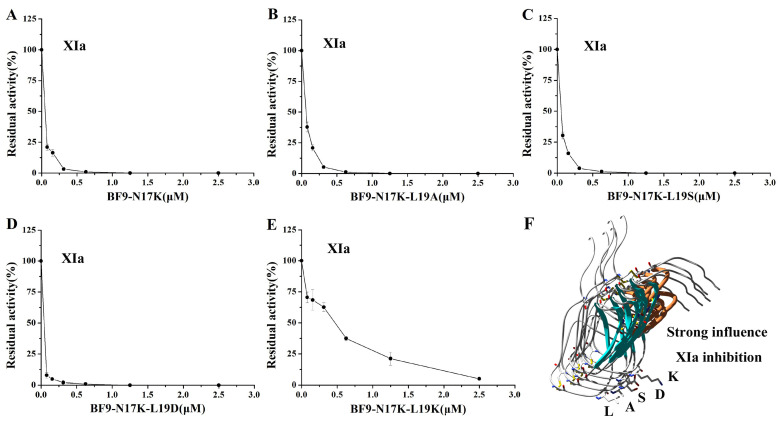
Serine protease XIa inhibitory activity of BF9-N17K and four designed analogues. (**A**) XIa inhibitory activity of BF9-N17K; (**B**) XIa inhibitory activity of BF9-N17K-L19A; (**C**) XIa inhibitory activity of BF9-N17K-L19S; (**D**) XIa inhibitory activity of BF9-N17K-L19D; (**E**) XIa inhibitory activity of BF9-N17K-L19K; (**F**) structural and XIa inhibitory function comparison between BF9-N17K and four designed analogues.

**Figure 4:**
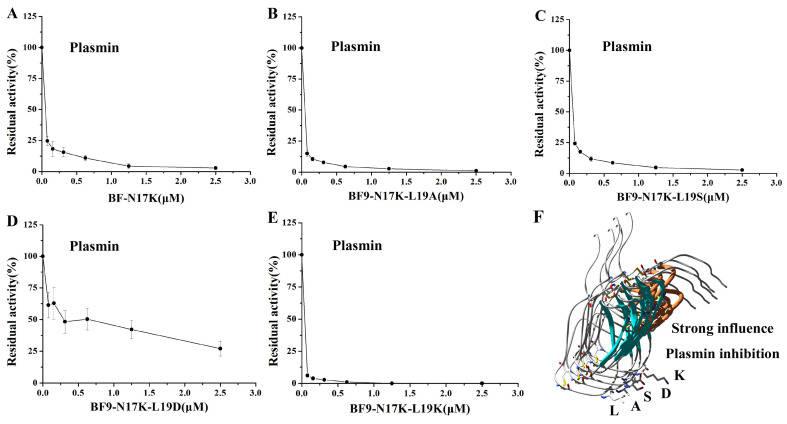
Serine protease plasmin inhibitory activity of BF9-N17K and four designed analogues. (**A**) Plasmin inhibitory activity of BF9-N17K; (**B**) plasmin inhibitory activity of BF9-N17K-L19A; (**C**) plasmin inhibitory activity of BF9-N17K-L19S; (**D**) plasmin inhibitory activity of BF9-N17K-L19D; (**E**) plasmin inhibitory activity of BF9-N17K-L19K; (**F**) structural and plasmin inhibitory function comparison between BF9-N17K and four designed analogues.

**Figure 5:**
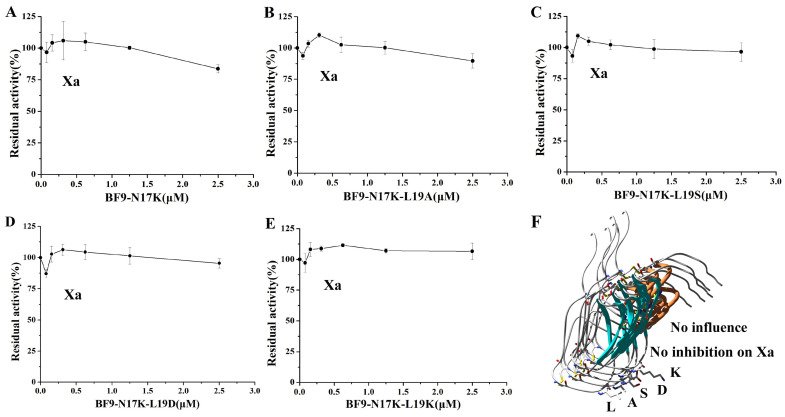
Serine protease Xa inhibitory activity of BF9-N17K and four designed analogues. (**A**) Xa inhibitory activity of BF9-N17K; (**B**) Xa inhibitory activity of BF9-N17K-L19A; (**C**) Xa inhibitory activity of BF9-N17K-L19S; (**D**) Xa inhibitory activity of BF9-N17K-L19D; (**E**) Xa inhibitory activity of BF9-N17K-L19K; (**F**) structural and Xa inhibitory function comparison between BF9-N17K and four designed analogues.

**Figure 6:**
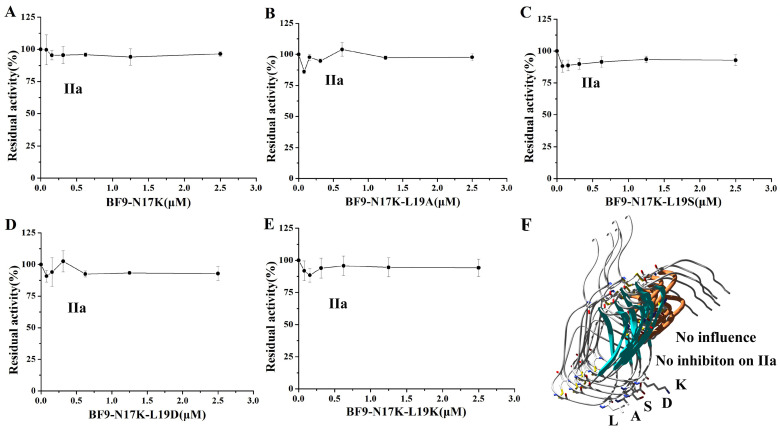
Serine protease IIa inhibitory activity of BF9-N17K and four designed analogues. (**A**) IIa inhibitory activity of BF9-N17K; (**B**) IIa inhibitory activity of BF9-N17K-L19A; (**C**) IIa inhibitory activity of BF9-N17K-L19S; (**D**) IIa inhibitory activity of BF9-N17K-L19D; (**E**) IIa inhibitory activity of BF9-N17K-L19K; (**F**) structural and IIa inhibitory function comparison between BF9-N17K and four designed analogues.

In our previous work, we designed an anticoagulant BF9-N17K that had significantly higher anticoagulation activity than the wild-type snake venom peptide BF9. However, the plasmin inhibitory activity of BF9-N17K was also very strong, with an IC_50_ of 16.33 ± 7.54 nM [24]. In this work, we obtained four new anticoagulants with similar intrinsic coagulation pathway inhibitory activities via P2′ site scanning. Whether there was a significant change in the inhibitory activity of the four new anticoagulants toward serine proteases remained to be studied. So, the XIa and other associated serine protease inhibitory activities of these four mutants were further evaluated. The enzyme inhibition activity experiments showed that BF9-N17K-L19A had apparent inhibitory activity toward XIa and plasmin, with IC_50_ values of 63.33 ± 5.31 nM and 7.00 ± 1.21 nM, respectively (Figure 3 and Figure S4), which are similar to those of the template peptide BF9-N17K and one analogue BF9-N17K-L19S. The similar serine protease inhibitory activities of three peptides, BF9-N17K, BF9-N17K-L19A, and BF9-N17K-L19S, were consistent with their similar anticoagulation activities (Figure 2), which suggested that the single-point mutation at the P2′ site with nonpolar amino acid A and polar amino acid S had minimal influences on the anticoagulation activity and serine protease inhibitory activity of the template Kunitz-type peptide BF9-N17K. Interestingly, when the P2′ site was replaced with charged amino acids, such as D and K, the situation was completely different (Figure 4). BF9-N17K-L19D showed higher XIa inhibitory activity than the template peptide BF9-N17K, and the inhibitory activity of BF9-N17K-L19D toward plasmin was also significantly reduced, with an IC_50_ of 459.33 ± 337.40 nM. However, the inhibitory activity of BF9-N17K-L19K toward plasmin was significantly enhanced, with an IC_50_ of 6.60 ± 3.40 nM, and its inhibitory activity toward XIa was reduced, with an IC_50_ of 321.67 ± 41.88 nM. Similar to the template peptide BF9-N17K, all the four P2′ site mutants had no inhibitory activity toward two classical anticoagulant targets, Xa and IIa (Figure 5 and Figure 6), which indicated that BF9-N17K is a good template for engineering selective XIa inhibitors, and the P2′ site amino acid classification scanning of BF9-N17K did not influence its insensitivity to the two classical anticoagulant targets Xa and IIa. In conclusion, these results indicated that through P2′ amino acid classification scanning with a snake venom Kunitz-type peptide scaffold, a new potent and selective XIa inhibitor, BF9-N17K-L19D, was discovered, which provides a new XIa-targeting lead drug for the development of new anticoagulants.

### 2.4. Structure and Function Relationship of BF9-N17K-L19D with XIa

**Figure 7:**
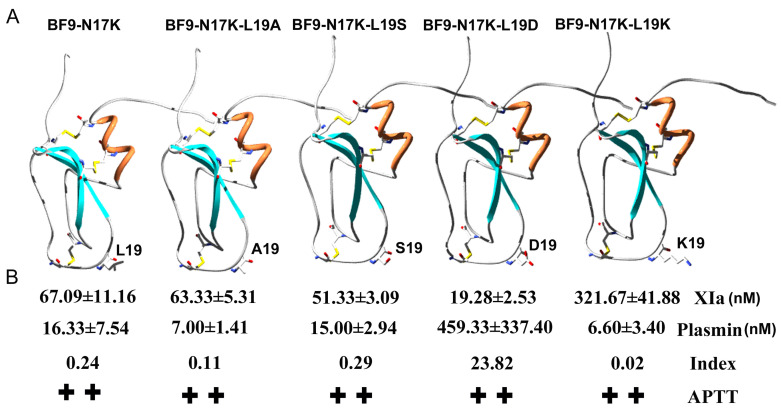
Serine proteases XIa and plasmin inhibitory activity comparison between BF9-N17K and four designed analogues. (**A**) The P2′ site comparison of BF9-N17K and four designed analogues; (**B**) XIa and plasmin inhibitory activity comparison of BF9-N17K and four designed analogues with different P2′ sites. ++ means strong anticoagulation activity in the APTT test.

Through P2′ amino acid classification scanning with a snake venom Kunitz-type peptide scaffold, we obtained a new potent and selective XIa inhibitor, BF9-N17K-L19D, with an IC_50_ of 19.28 ± 2.53 nM. In order to further characterize its structure and function relationship and its selectivity toward XIa, the XIa and plasmin inhibitory activity of five Kunitz-type peptides, BF9-N17K, BF9-N17K-L19A, BF9-N17K-L19S, BF9-N17K-L19D, and BF9-N17K-L19K were compared (Figure 7). The results showed that BF9-N17K, BF9-N17K-L19A, and BF9-N17K-L19S had similar inhibitory activities toward XIa and plasmin, but BF9-N17K-L19D had enhanced inhibitory activity toward XIa, and BF9-N17K-L19K had enhanced inhibitory activity toward plasmin. The inhibitory activity profile of BF9-N17K-L19D and its analogues suggested that the characteristics of the charge of the P2′ site might be associated with the drug selectivity between the anticoagulant target XIa and hemostatic agent target plasmin. The noncharged amino acid at the P2′ site might have similar effects on XIa and plasmin inhibitory activity, such as the polar amino acid S and nonpolar amino acids A and L. Our results showed that the replacements with charged amino acid might be an effective strategy to enhance drug selectivity toward the coagulation factor XIa and the hemostatic target plasmin [17,23,26,30].

## 3. Discussion

The development of new anticoagulants to prevent pathologic thrombi has enormous clinical application value [33]. The success of anticoagulants targeting Xa and IIa has indicated that research on specific inhibitors is important for the development of new anticoagulants [34,35,36]. Before our work, several potent XIa inhibitors with Kunitz-type scaffold have been found in venomous animals, blood-sucking animals, and the human body; through artificial design; and from other resources [37,38,39].

Through P2′ site amino acid classification scanning, we enhanced the selectivity of a reported XIa inhibitor, BF9-N17K, successfully, suggesting that molecular design is a useful strategy to optimize Kunitz-type peptide [40]. Before our work, using the natural XIa inhibitor Fasxiator as Kunitz-type peptide template, Kang’s research team from Singapore designed a mutant peptide Fasxiator_N17R, L19E_ with an IC_50_ of about 1 nM toward XIa and a selectivity over 100-fold higher towards plasmin, which showed a good balance between potency and selectivity [17]. With the natural XIa inhibitor PN2KPI as a Kunitz-type peptide template, William P. Sheffielda’s research team from Canada designed a mutant peptide PN2KPI-M17D, which also had high selectivity toward XIa [19,23,27,41]. These studies indicated that the acidic amino acids D and E at the P2′ site might favor potent and selective inhibitory activity toward the new anticoagulant target XIa. These data are also consistent with our present work in that BF9-N17K-L19D had better selectivity toward XIa than the template peptide BF9-N17K and three analogues BF9-N17K-L19A, BF9-N17K-L19S, and BF9-N17K-L19K. This might be a new clue regarding XIa-inhibitory macrocyclic-peptide and small-molecule mimics, especially for the P2′ site [42,43,44].

**Figure 8:**
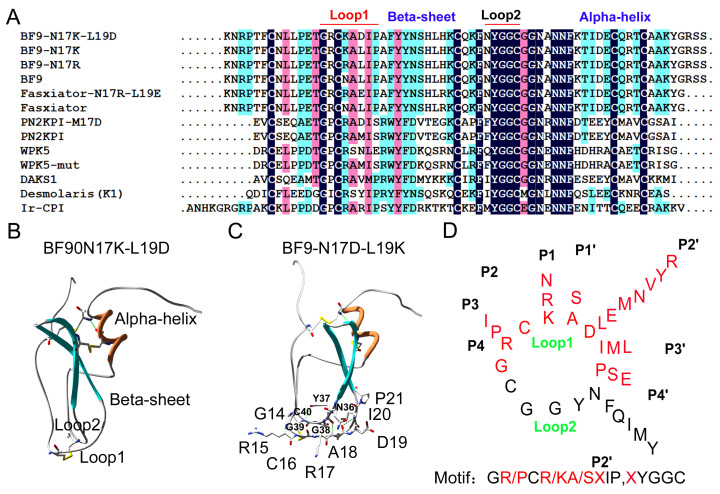
Molecular diversity and a common XIa-inhibitory motif of XIa inhibitors with Kunitz-type structural fold. (**A**) Amino acid sequence alignments of XIa inhibitors with Kunitz-type structural fold; (**B**) potential functional loops 1 and 2 of BF9-N17K-L19D; (**C**) detailed residues of functional loops 1 and 2 of BF9-N17K-L19D; (**D**) a common motif GR/PCR/KA/SIP-XYGGC of XIa inhibitors with Kunitz-type structural fold, where X means any 1 of the 20 amino acids.

In addition to the P2′ site, the other sites in Kunitz-type peptides might also contribute the potency and selectivity toward XIa [21,25,43]. In order to explore a possible common motif of Kunitz-type peptides regarding XIa inhibitory activity, the reported XIa inhibitors with a Kunitz-type structural fold were collected and analyzed. The sequence alignments showed that the sequences in loop 1 and loop 2 of these XIa-inhibitory Kunitz-type peptides were conserved, and the sequences located in the alpha-helix and beta-sheet regions were not conserved [13,14,17,20,21,22,24] (Figure 8). Based on the sequence characteristics, a common motif GR/PCR/KA/SXIP-XYGGC is proposed according to these reported XIa-inhibitory Kunitz-type peptides and our present work. Based on the common motif of XIa-inhibitory Kunitz-type peptides, two X sites in the motif might be the major sites that can be engineered for enhancing drug selectivity and maintaining potent drug activity toward XIa, and the other eleven sites might provide only very limited choices for XIa inhibitor design. This might also be a new clue regarding the potency and selectivity of XIa inhibitors, not only for the designing peptides, but also for mimicking macrocyclic peptides and small molecules [42,43,44,45].

## 4. Conclusions

In conclusion, with a snake venom Kunitz-type scaffold as a template, we designed a potent and selective XIa inhibitor, BF9-N17K-L19D, via the P2′ site amino acid classification scanning strategy, highlighting that two acidic amino acids, D and E, might be suitable choices for the P2′ site of selective XIa inhibitors with a Kunitz-type scaffold. In addition, a common motif, GR/PCR/KA/SXIP-XYGGC, was proposed for the XIa-inhibitory Kunitz-type peptides, which might provide a new clue for further peptide engineering.

## 5. Materials and Methods

### 5.1. Peptide Design, Recombinant Plasmids, and Recombinant Peptides

The P2′ site of BF9-N17K was optimally designed considering different amino acid types, and the natural site L19 was mutated into nonpolar aliphatic amino acids (alanine), polar neutral amino acids (serine), acidic amino acids (aspartate), and alkaline amino acids (lysine). Four recombinant plasmids, BF9-N17K-L19A, BF9-N17K-L19S, BF9-N17K-L19D, and BF9-N17K-L19K, were constructed by GenScript (Nanjing, China). Four recombinant peptides were expressed and purified using similar methods with the template peptide BF9-N17K [16,24,39,46]. The atomic structures of all Kunitz-domain peptides were modeled using the SWISS-MODEL server (https://www.swissmodel.expasy.org/, accessed on 9 November 2024).

### 5.2. Serine Protease Inhibitory Activity Assay

Using the fluorogenic substrate method the coagulation factors (FXIa, FXa, and FIIa) and fibrinolytic enzyme (plasmin), which play a key role in the coagulation process, were detected as we have described before [16,24,39,46]. The XIa-selective anticoagulation peptides with better anticoagulation activity were screened. The experiment was repeated three times, and the results are presented the mean and standard deviation (SD) of quadruplicate tests. The curve of serine protease inhibitory activity was generated in SigmaPlot 12.5 software, and the IC_50_ was calculated according to the following equation (four-parameter logistic curve): y = min + (max − min)/(1 + (x/IC_50_) − Hillslope).

### 5.3. Anticoagulation Activity Assay

Partial thromboplastin time (APTT), prothrombin time (PT), and thrombin time (TT) of 4 mutant peptides were detected by the turbidity method following the method that we have described before [16,24,39,46], and the anticoagulant function of the peptides with better anticoagulation function was screened. The experiment was repeated three times, and the results are presented as the mean and standard deviation (SD) of quadruplicate tests.

## Data Availability

The original contributions presented in this study are included in this article and Supplementary Material. Further inquiries can be directed to the corresponding author.

## Supplementary Materials

The following supporting information can be downloaded at: https://www.mdpi.com/article/10.3390/toxins17010023/s1, Figure S1. Purification of BF9-N17K and four designed analogues by high-performance liquid chromatography (HPLC). A, Purification of peptide BF9-N17K by HPLC; B, purification of peptide BF9-N17K-L19A by HPLC; C, purification of peptide BF9-N17K-L19S by HPLC; D, purification of peptide BF9-N17K-L19D by HPLC; E, purification of peptide BF9-N17K-L19K by HPLC. Figure S2. Extrinsic coagulation pathway inhibitory activity of BF9-N17K and four designed analogues tested via PT experiments. A, Extrinsic coagulation pathway inhibitory activity of BF9-N17K; B, extrinsic coagulation pathway inhibitory activity of BF9-N17K-L19A; C, extrinsic coagulation pathway inhibitory activity of BF9-N17K-L19S; D, extrinsic coagulation pathway inhibitory activity of BF9-N17K-L19D; E, extrinsic coagulation pathway inhibitory activity of BF9-N17K-L19K; F, structural and extrinsic coagulation pathway inhibitory function comparison of BF9-N17K and four designed analogues. Figure S3. Common coagulation pathway inhibitory activity of BF9-N17K and four designed analogues tested through TT experiments. A, Common coagulation pathway inhibitory activity of BF9-N17K; B, common coagulation pathway inhibitory activity of BF9-N17K-L19A; C, common coagulation pathway inhibitory activity of BF9-N17K-L19S; D, common coagulation pathway inhibitory activity of BF9-N17K-L19D; E, common coagulation pathway inhibitory activity of BF9-N17K-L19K; F, structural and common coagulation pathway inhibitory function comparison of BF9-N17K and four designed analogues. Figure S4. Serine protease XIa inhibitory activity difference of BF9-N17K and four designed analogues. A, Serine protease XIa inhibitory activity of BF9-N17K with low concentration peptides. B, Serine protease XIa inhibitory activity of BF9-N17K-L19D with low concentration peptides. C, The comparison of XIa inhibitory activities of BF9-N17K and four designed analogues with their IC_50_ values.

## References

[1] Kaiser R., Dewender R., Mulkers M., Stermann J., Rossaro D., Di Fina L., Li L., Gold C., Schmid M., Kaab L. (2024). Procoagulant platelet activation promotes venous thrombosis. Blood.

[2] Seanoon K., Payongsri P., Vivithanaporn P., Sirachainan N., Chuansumrit A., Hongeng S., Tanratana P. (2022). Mutations of TFPI-binding exosites on factor VII cause bleeding phenotypes in factor VII deficiency. Blood Adv..

[3] Naudin C., Burillo E., Blankenberg S., Butler L., Renne T. (2017). Factor XII Contact Activation. Semin. Thromb. Hemost..

[4] Galli M., Gragnano F., Berteotti M., Marcucci R., Gargiulo G., Calabro P., Terracciano F., Andreotti F., Patti G., De Caterina R. (2024). Antithrombotic Therapy in High Bleeding Risk, Part II: Noncardiac Percutaneous Interventions. JACC. Cardiovasc. Interv..

[5] Galli M., Gragnano F., Berteotti M., Marcucci R., Gargiulo G., Calabro P., Terracciano F., Andreotti F., Patti G., De Caterina R. (2024). Antithrombotic Therapy in High Bleeding Risk, Part I: Percutaneous Cardiac Interventions. JACC. Cardiovasc. Interv..

[6] Frunt R., El Otmani H., Smits S., Clark C.C., Maas C. (2024). Factor XII contact activation can be prevented by targeting 2 unique patches in its epidermal growth factor-like 1 domain with a nanobody. J. Thromb. Haemost. JTH.

[7] Heestermans M., Naudin C., Mailer R.K., Konrath S., Klaetschke K., Jamsa A., Frye M., Deppermann C., Pula G., Kuta P. (2021). Identification of the factor XII contact activation site enables sensitive coagulation diagnostics. Nat. Commun..

[8] Puy C., Moellmer S.A., Pang J., Vu H.H., Melrose A.R., Lorentz C.U., Tucker E.I., Shatzel J.J., Keshari R.S., Lupu F. (2024). Coagulation factor XI regulates endothelial cell permeability and barrier function in vitro and in vivo. Blood.

[9] Moellmer S.A., Puy C., McCarty O.J.T. (2024). Biology of factor XI. Blood.

[10] Gailani D., Gruber A. (2024). Targeting factor XI and factor XIa to prevent thrombosis. Blood.

[11] Harrington J., Piccini J.P., Alexander J.H., Granger C.B., Patel M.R. (2023). Clinical Evaluation of Factor XIa Inhibitor Drugs: JACC Review Topic of the Week. J. Am. Coll. Cardiol..

[12] Xie Z., Meng Z., Yang X., Duan Y., Wang Q., Liao C. (2023). Factor XIa Inhibitors in Anticoagulation Therapy: Recent Advances and Perspectives. J. Med. Chem..

[13] Decrem Y., Rath G., Blasioli V., Cauchie P., Robert S., Beaufays J., Frere J.M., Feron O., Dogne J.M., Dessy C. (2009). Ir-CPI, a coagulation contact phase inhibitor from the tick Ixodes ricinus, inhibits thrombus formation without impairing hemostasis. J. Exp. Med..

[14] Ma D., Mizurini D.M., Assumpcao T.C., Li Y., Qi Y., Kotsyfakis M., Ribeiro J.M., Monteiro R.Q., Francischetti I.M. (2013). Desmolaris, a novel factor XIa anticoagulant from the salivary gland of the vampire bat (Desmodus rotundus) inhibits inflammation and thrombosis in vivo. Blood.

[15] Ranasinghe S., McManus D.P. (2013). Structure and function of invertebrate Kunitz serine protease inhibitors. Dev. Comp. Immunol..

[16] Ding L., Hao J., Luo X., Zhu W., Wu Z., Qian Y., Hu F., Liu T., Ruan X., Li S. (2018). The Kv1.3 channel-inhibitory toxin BF9 also displays anticoagulant activity via inhibition of factor XIa. Toxicon Off. J. Int. Soc. Toxinol..

[17] Chen W., Carvalho L.P., Chan M.Y., Kini R.M., Kang T.S. (2015). Fasxiator, a novel factor XIa inhibitor from snake venom, and its site-specific mutagenesis to improve potency and selectivity. J. Thromb. Haemost. JTH.

[18] Yang W., Feng J., Wang B., Cao Z., Li W., Wu Y., Chen Z. (2014). BF9, the first functionally characterized snake toxin peptide with Kunitz-type protease and potassium channel inhibiting properties. J. Biochem. Mol. Toxicol..

[19] Navaneetham D., Jin L., Pandey P., Strickler J.E., Babine R.E., Abdel-Meguid S.S., Walsh P.N. (2005). Structural and mutational analyses of the molecular interactions between the catalytic domain of factor XIa and the Kunitz protease inhibitor domain of protease nexin 2. J. Biol. Chem..

[20] Wu W., Li H., Navaneetham D., Reichenbach Z.W., Tuma R.F., Walsh P.N. (2012). The kunitz protease inhibitor domain of protease nexin-2 inhibits factor XIa and murine carotid artery and middle cerebral artery thrombosis. Blood.

[21] Zheng Y.Z., Ji X.R., Liu Y.Y., Jiang S., Yu X.Y., Jia Z.P., Zhao Y., Zhang J.Q., Zhang J.L., Kong Y. (2021). WPK5, a Novel Kunitz-Type Peptide from the Leech Whitmania pigra Inhibiting Factor XIa, and Its Loop-Replaced Mutant to Improve Potency. Biomedicines.

[22] Jia Z., Liu Y., Ji X., Zheng Y., Li Z., Jiang S., Li H., Kong Y. (2021). DAKS1, a Kunitz Scaffold Peptide from the Venom Gland of Deinagkistrodon acutus Prevents Carotid-Artery and Middle-Cerebral-Artery Thrombosis via Targeting Factor XIa. Pharmaceuticals.

[23] Eltringham-Smith L.J., Bhakta V., Sheffield W.P. (2021). Selection and in vitro and in vivo characterization of a Kunitz protease inhibitor domain of protease nexin 2 variant that inhibits factor XIa without inhibiting plasmin. J. Biotechnol..

[24] Ding L., Hao J., Luo X., Chen Z. (2018). Engineering varied serine protease inhibitors by converting P1 site of BF9, a weakly active Kunitz-type animal toxin. Int. J. Biol. Macromol..

[25] Sun F., Wang W., Li Z., Li Y., Guo W., Kong Y. (2024). Design, expression and biological evaluation of DX-88mut as a novel selective factor XIa inhibitor for antithrombosis. Bioorg. Chem..

[26] Navaneetham D., Wu W., Li H., Sinha D., Tuma R.F., Walsh P.N. (2013). P1 and P2′ site mutations convert protease nexin-2 from a factor XIa inhibitor to a plasmin inhibitor. J. Biochem..

[27] Su Y.C., Miller T.N., Navaneetham D., Schoonmaker R.T., Sinha D., Walsh P.N. (2011). The role of factor XIa (FXIa) catalytic domain exosite residues in substrate catalysis and inhibition by the Kunitz protease inhibitor domain of protease nexin 2. J. Biol. Chem..

[28] Kumar Y., Vadivel K., Schmidt A.E., Ogueli G.I., Ponnuraj S.M., Rannulu N., Loo J.A., Bajaj M.S., Bajaj S.P. (2014). Decoy plasminogen receptor containing a selective Kunitz-inhibitory domain. Biochemistry.

[29] Bajaj M.S., Ogueli G.I., Kumar Y., Vadivel K., Lawson G., Shanker S., Schmidt A.E., Bajaj S.P. (2011). Engineering kunitz domain 1 (KD1) of human tissue factor pathway inhibitor-2 to selectively inhibit fibrinolysis: Properties of KD1-L17R variant. J. Biol. Chem..

[30] Salameh M.A., Soares A.S., Hockla A., Radisky D.C., Radisky E.S. (2011). The P(2)′ residue is a key determinant of mesotrypsin specificity: Engineering a high-affinity inhibitor with anticancer activity. Biochem. J..

[31] Ding L., Wang X., Liu H., San M., Xu Y., Li J., Li S., Cao Z., Li W., Wu Y. (2015). A new Kunitz-type plasmin inhibitor from scorpion venom. Toxicon Off. J. Int. Soc. Toxinol..

[32] Chen Z.Y., Hu Y.T., Yang W.S., He Y.W., Feng J., Wang B., Zhao R.M., Ding J.P., Cao Z.J., Li W.X. (2012). Hg1, novel peptide inhibitor specific for Kv1.3 channels from first scorpion Kunitz-type potassium channel toxin family. J. Biol. Chem..

[33] Mega J.L., Simon T. (2015). Pharmacology of antithrombotic drugs: An assessment of oral antiplatelet and anticoagulant treatments. Lancet.

[34] Straub A., Roehrig S., Hillisch A. (2011). Oral, direct thrombin and factor Xa inhibitors: The replacement for warfarin, leeches, and pig intestines?. Angewandte Chemie.

[35] Perzborn E., Roehrig S., Straub A., Kubitza D., Misselwitz F. (2011). The discovery and development of rivaroxaban, an oral, direct factor Xa inhibitor. Nature reviews. Drug Discov..

[36] Desai U.R. (2004). New antithrombin-based anticoagulants. Med. Res. Rev..

[37] Jmel M.A., Voet H., Araujo R.N., Tirloni L., Sa-Nunes A., Kotsyfakis M. (2023). Tick Salivary Kunitz-Type Inhibitors: Targeting Host Hemostasis and Immunity to Mediate Successful Blood Feeding. Int. J. Mol. Sci..

[38] Liu Y., Jiang S., Li Q., Kong Y. (2021). Advances of Kunitz-type serine protease inhibitors. Sheng Wu Gong Cheng Xue Bao = Chin. J. Biotechnol..

[39] Sun F., Deng X., Gao H., Ding L., Zhu W., Luo H., Ye X., Luo X., Chen Z., Qin C. (2024). Characterization of Kunitz-Domain Anticoagulation Peptides Derived from Acinetobacter baumannii Exotoxin Protein F6W77. Toxins.

[40] Simeon R., Chen Z. (2018). In vitro-engineered non-antibody protein therapeutics. Protein Cell.

[41] Navaneetham D., Sinha D., Walsh P.N. (2010). Mechanisms and specificity of factor XIa and trypsin inhibition by protease nexin 2 and basic pancreatic trypsin inhibitor. J. Biochem..

[42] Martian P.C., Tertis M., Leonte D., Hadade N., Cristea C., Crisan O. (2025). Cyclic peptides: A powerful instrument for advancing biomedical nanotechnologies and drug development. J. Pharm. Biomed. Anal..

[43] Ji X., Nielsen A.L., Heinis C. (2024). Cyclic Peptides for Drug Development. Angew. Chem..

[44] Imai M., Colas K., Suga H. (2024). Protein Grafting Techniques: From Peptide Epitopes to Lasso-Grafted Neobiologics. ChemPlusChem.

[45] Vinogradov A.A., Yin Y., Suga H. (2019). Macrocyclic Peptides as Drug Candidates: Recent Progress and Remaining Challenges. J. Am. Chem. Soc..

[46] Ding L., Shu Z., Hao J., Luo X., Ye X., Zhu W., Duan W., Chen Z. (2022). Schixator, a new FXa inhibitor from Schistosoma japonicum with antithrombotic effect and low bleeding risk. Biochem. Biophys. Res. Commun..

