# Supplementary material for: Improvement in XIa Selectivity of Snake Venom Peptide Analogue BF9-N17K Using P2′ Amino Acid Replacements
Source: Toxins (Basel). 2025 Jan 5;17(1):23. doi: 10.3390/toxins17010023 (PMC11769409; doi:10.3390/toxins17010023)
Supplement: Supplementary file 1 [file toxins-17-00023-s001.zip › toxins-3360895-supplementary.pdf]

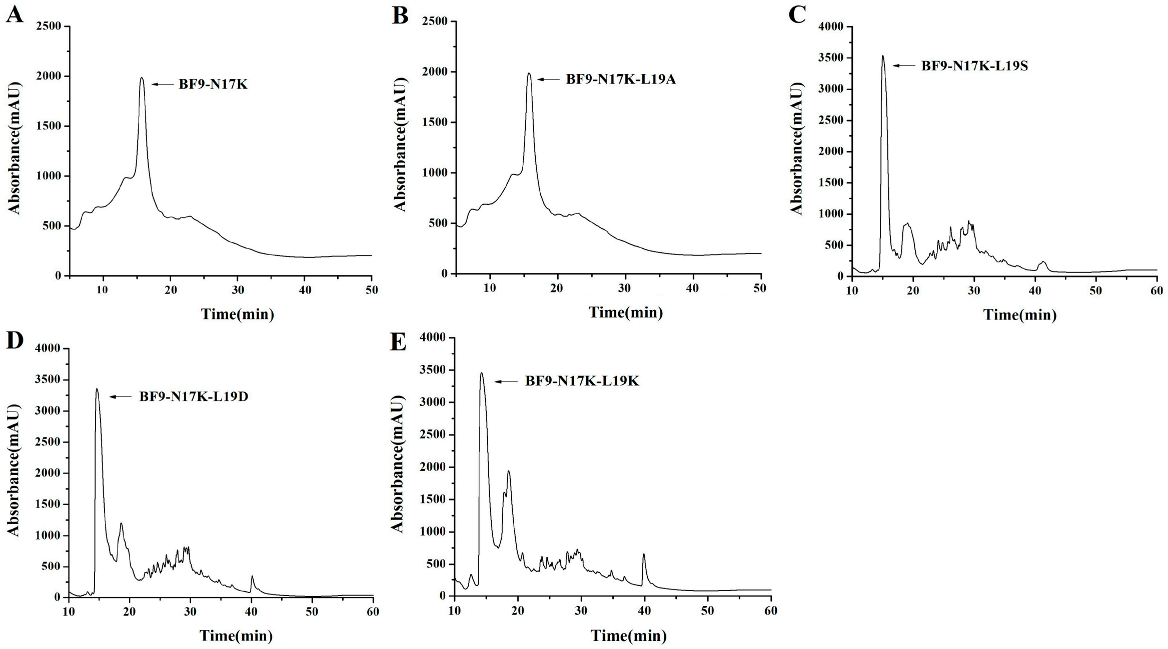

Figure S1. Purification of BF9-N17K and four designed analogues by high-performance liquid chromatography (HPLC). A, Purification of peptide BF9-N17K by HPLC; B, purification of peptide BF9-N17K-L19A by HPLC; C, purification of peptide BF9-N17K-L19S by HPLC; D, purification of peptide BF9-N17K-L19D by HPLC; E, purification of peptide BF9-N17K-L19K by HPLC.

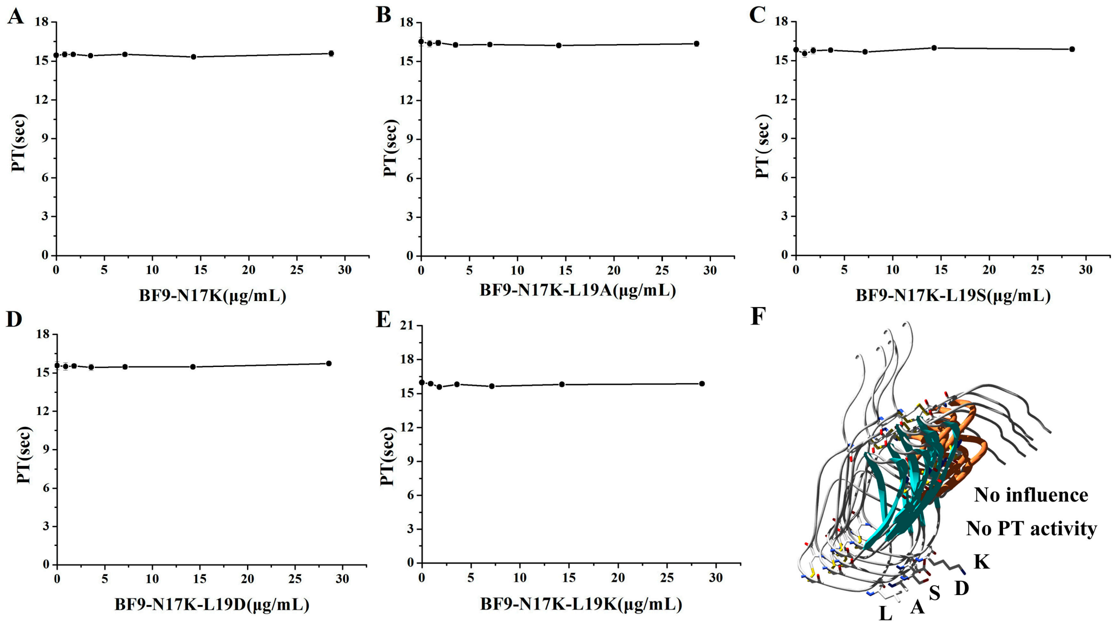

Figure S2. Extrinsic coagulation pathway inhibitory activity of BF9-N17K and four designed analogues tested via PT experiments. A, Extrinsic coagulation pathway inhibitory activity of BF9-N17K; B, extrinsic coagulation pathway inhibitory activity of BF9-N17K-L19A; C, extrinsic coagulation pathway inhibitory activity of BF9-N17K-L19S; D, extrinsic coagulation pathway inhibitory activity of BF9-N17K-L19D; E, extrinsic coagulation pathway inhibitory activity of BF9-N17K-L19K; F, structural and extrinsic coagulation pathway inhibitory function comparison of BF9-N17K and four designed analogues.

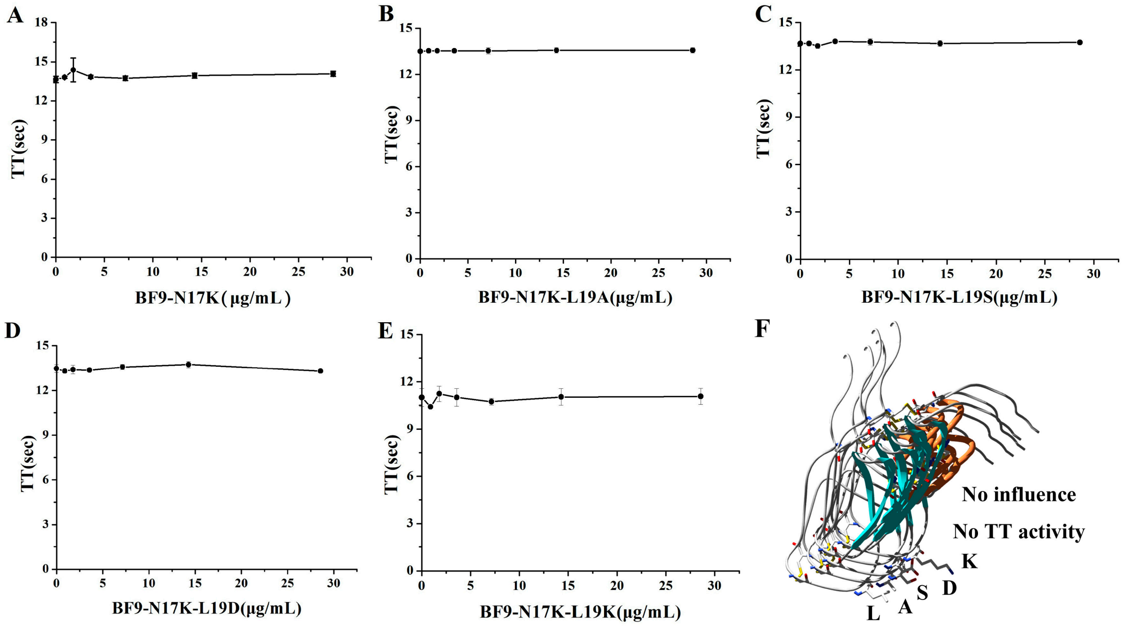

Figure S3. Common coagulation pathway inhibitory activity of BF9-N17K and four designed analogues tested through TT experiments. A, Common coagulation pathway inhibitory activity of BF9-N17K; B, common coagulation pathway inhibitory activity of BF9-N17K-L19A; C, common coagulation pathway inhibitory activity of BF9-N17K-L19S; D, common coagulation pathway inhibitory activity of BF9-N17K-L19D; E, common coagulation pathway inhibitory activity of BF9-N17K-L19K; F, structural and common coagulation pathway inhibitory function comparison of BF9-N17K and four designed analogues.

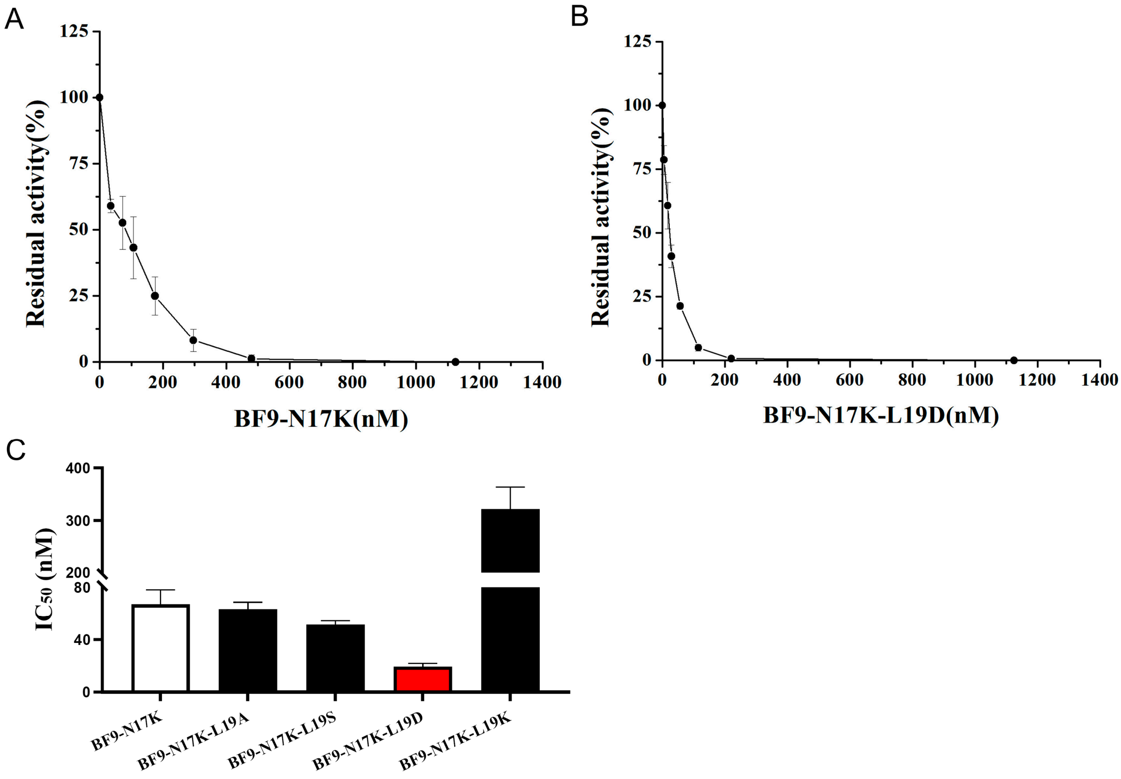

Figure S4. Serine protease XIa inhibitory activity difference of BF9-N17K and four designed analogues. A, Serine protease XIa inhibitory activity of BF9-N17K with low concentration peptides.

B, Serine protease XIa inhibitory activity of BF9-N17K-L19D with low concentration peptides. C, The comparison of XIa inhibitory activities of BF9-N17K and four designed analogues with their IC_{50} values.
